# Randomised trial of two embolic agents for uterine artery embolisation for fibroids: Gelfoam versus Embospheres (RAGE trial)

**DOI:** 10.1186/s42155-018-0044-y

**Published:** 2019-01-08

**Authors:** R. Yadavali, G. Ananthakrishnan, M. Sim, K. Monaghan, G. McNaught, I. Hamoodi, F. Bryden, S. Lassman, J. G. Moss

**Affiliations:** 1 Institute of Cardiovascular and Medical Sciences, BHF Glasgow Cardiovascular Research Centre, 126 University Place, Glasgow, G12 8TA UK; 2 School of Mathematics and Statistics, University of Glasgow, Glasgow, G12 8QW UK; 3 Clinical Research Imaging Centre, University of Edinburgh, Queen’s Medical Research Institute, 47 Little France Crescent, Edinburgh, EH16 4TJ UK; 4 University Department of Obstetrics and Gynaecology, Glasgow, New Lister Building, 10-16 Alexandra Parade, Glasgow, G31 2ER UK; 5 Radiology department, Stobhill ACH Hospital, 133 Balornock Road, Glasgow, G21 3UW UK; 6 Radiology department, Gartnavel General Hospital, 1053 Great Western Rd, Glasgow, G12 OYN UK

## Abstract

**Background:**

Uterine artery embolisation (UAE) is an established treatment option for women with symptomatic uterine fibroids who wish to avoid surgery. However the most efficacious embolic agent remains uncertain.

**Methods:**

We conducted a pilot double blind randomized controlled trial comparing Gelfoam with Embospheres in women undergoing UAE. Outcomes recorded at baseline, 24-h, 1 and 6 months included complications, inflammatory, haematological markers and ovarian function. Contrast enhanced MRI (CEMRI) was acquired at baseline, 24-h and 6 months. Pain score (visual analogue) was measured at 24-h, quality of life (UFS-Qol) at baseline, 1 and 6 months. All patients were followed to 6 months.

**Results:**

Twenty patients were randomized 1:1 to receive either Gelfoam (G) or Embospheres (E). The UFS-Qol symptom score improved in both groups at 6 months mean of 64 ± 18 to 23 ± 16 and 54 ± 15 to 32 ± 26 in the E and G groups respectively. UFS-Qol HRQL also improved in both groups at 6 months, mean 41 ± 28 to 79 ± 20 and 53 ± 19 to 78 ± 21 in the E and G groups respectively.

Uterine volume at 6 months reduced from 1018 ± 666mls to 622 ± 436 (*p* = 0.001) and from 1026 ± 756 to 908 ± 720 (*p* = 0.15) in the E and G groups respectively. There was a significant difference between groups for this parameter *p* = 0.01. All uterine arteries were patent at 24-h and 6 months. Complete (100%) fibroid infarction rates were 5(50%) and 2(20%) in the E and G groups respectively. None of the other outcome measures showed any between group differences. There were no re-interventions in either group.

**Conclusion:**

The only significant between group differences was for a greater reduction in uterine volume at 6 months in the E group. A larger trial (estimate 172 subjects) is required to determine whether other apparent differences are clinically and statistically significant.

**Trial registration:**

ISRCTN67347987

## Introduction

Uterine fibroids are the most common tumour in women (Wilcox et al., 1994). When symptomatic UAE is now an established treatment option for those who wish to avoid conventional surgery (Edwards et al., 2007; Moss et al., 2011). An increasing number of embolic agents are used and licensed for UAE which include so called “temporary agents” such as Gelfoam (Ethicon Medical Ltd. Livingston, Scotland) and more permanent options such as Polyvinyl alcohol (PVA) particles ((Contour, Boston Scientific, Hemel Hempstead, UK; PVA (Cook Medical, Bloomington, IN, USA)), Trisacrylgelatin microsphere (Embospheres, Merit Medical Ltd., Coatbridge, Scotland), PVA hydrogel beads with acrylic polymer (Beadblock, Terumo UK Ltd., Surrey, UK.), HydroPearl microspheres (Terumo UK Ltd., Surrey UK) and Hydrogel microspheres coated with Polyzene-F (Embozene, Celonova BioSciences, Basingstoke, UK).

Gelfoam is rapidly reabsorbed by the body and is the agent of choice for controlling obstetric haemorrhage. In theory at least it should also be suitable for fibroids and Japanese workers have demonstrated its efficacy reporting cumulative rates of symptom control of 96.9% at 1 year, 89.5% at 3 years, and 89.5% at 5 years respectively (Das et al., 2014). In spite of this Gelfoam has not gained widespread acceptance for fibroid embolisation. There are 5 randomised trials comparing some of these products (predominantly Embospheres) but there is little consensus as to which is the most effective from an efficacy, safety clinical or health economic perspective (Spies et al., 2002). There has been no attempt to compare Gelfoam (the cheapest and most established) with any of the other products.

The aim of this small pilot study was to collect a wide range of biochemical, imaging, and clinical parameters to help inform a larger trial.

## Methods

The study was conducted in one large tertiary referral hospital in the U.K. Patients were randomized between March 2011 and January 2012 and 6 month follow up was complete in August 2012.

The study was approved by the regional ethics committee (West of Scotland REC ref. 09/S0703/108) and funded by a research grant from the British Society of Interventional Radiology. Patients were recruited and followed up at an Interventional Radiology clinic. A trial coordinator (MS) managed the trial.

### Patients

Women at least 18 years old were eligible if they had one or more symptomatic fibroids of more than 2 cm diameter confirmed by MRI. Patients with subserosal pedunculated fibroids (stalk base < 1 cm), severe allergy to iodinated contrast media, contraindication to contrast enhanced magnetic resonance imaging (CEMRI), recent or ongoing pelvic infection, and the presence of significant other pelvic pathology e.g. adenomyosis were excluded from the study. Patients were blinded to the treatment allocation.

### Procedures

Patients were randomly assigned by the trial co-ordinator using a computer-generated schedule on a 1:1 basis. There was no stratification or pre-specified subgroups.

UAE was carried out by one of three experienced interventional radiologists (JGM, RY, AG) using a standard technique. Using a right common femoral artery access each uterine artery was selected with a 2.7 French co-axial catheter (Progreat, Terumo UK Ltd., Surrey, UK.) and embolisation carried out on each side to complete stasis. Complete stasis was defined as no flow observed in the main uterine artery over a 60 s observation period. No attempt was made to visualize or embolise the ovarian arteries as is our normal practice with the primary procedure.

Spongostan Absorbable Haemostatic Gelatin Sponge (Ethicon Medical Ltd. Livingston, Scotland) was manually cut into approximately 1 mm squares and mixed with iodinated contrast (Omnipaque 300) to form a ‘slurry’ using a 5-10 cc reservoir syringe and a 1-2 cc delivery syringe connected via a three way tap. The residual sponge was weighed to quantify the amount used.

Embospheres (Merit Medical Ltd., Coatbridge, Scotland) were used according to the manufacturer instruction for use namely mixed with an equal volume of contrast and the first two ampoules on each side being 500–700 μm followed with 700–900 μm if necessary.

Pain was managed using the existing hospital protocol, which included opioids, and other medication included midazolam and anti-emetics. All patients were admitted overnight after the procedure.

### Outcome measures

The outcome measures and time of assessment are summarized in Table 1.

**Table 1:** Time points for all outcome measures

Parameter	Baseline	24 h	1 month	6 months
UFS-Qol	x			x
Pain score (VAS)		x		
CE MRI	x	x		x
Sex hormones AMH	x	x	x	x
Inflammatory markers (WCC & CRP)	x	x	x	x
Complications	x	x	x	x

In summary outcomes were measured at baseline, 1 and 6 months. All blood tests were additionally measured at 24 h prior to hospital discharge. Quality of life (UFSQoL) was assessed at baseline and 6 months.

Pain scores at 24 h were recorded on a standard visual analogue scale ranging from 0 (no pain) to 10 (worst pain). UFS-Qol comprised a symptom severity score and a health related quality of life (HRQL) score (van Rooij et al., 2005). Transformed scores (0–100) were then calculated using a formula for each of symptom severity and HRQL. Higher symptom scores indicate greater symptom severity and higher HRQL scores indicate better quality of life.

Inflammatory markers comprised the white cell count (WCC) and C-reactive Protein (CRP) measurements.

Sex hormones included follicle stimulating hormone (FSH), leutinising hormone (LH) and estradiol (E2). Anti-mullerian hormone (AMH) is a marker for ovarian follicle reserve measured in pmol/L (Beckman Coulter AMH Generation II Elisa method) (Sacks et al., 2003). These were all measured at baseline, 24 h, 1 and 6 months.

### Imaging

All patients underwent CEMRI on a 1.5 T HDx MR system with an 8 channel surface coil (GE Cardiac coil) positioned over the pelvis. Images and spectra of the dominant fibroid were acquired at three time points: baseline pre-UAE, 24-h and 6 months post-UAE.

T2 weighted Fast Recovery Fast Spin Echo (FRFSE) images acquired in the sagittal and coronal planes (TR 4960 ms/TE 85 ms/16, FOV 240 mm, 256 × 384 matrix, slice thickness 4 mm, echo train length 16). These sequences were used for planning the spectroscopy acquisition and volume measurements. Sagittal T1 weighted images were acquired using the liver acquisition and volume acquisition (LAVA) sequence (TR/TE = 4.72 ms/2.32 ms, FOV/matrix/slice thickness = 280 mm/320 × 192/5 mm) before and after injection of gadolinium contrast agent (Gadovist, Bayer plc., Berkshire, UK). A standard magnetic resonance angiogram sequence of the uterine arteries was acquired following contrast enhancement. Some of the more complex imaging data (MR spectroscopy and diffusion weighted imaging) has been published separately (Katsumori et al., 2006).

Two diagnostic radiologists blinded to the treatment allocation interpreted the images independently. Any disagreements were resolved by consensus. Imaging data extracted included dominant fibroid diameter and uterine volume, uterine artery patency (occluded or patent) and fibroid infarction. Fibroid infarction assessment used the entire fibroid collection and a simple visual scoring system (or eyeballing) used to put the patient into one of three groups: complete (100%), almost complete (99–90%) and incomplete (< 90%). Patency referred to the main uterine artery as this was the only part of the vessel reliably imaged on MR.

Complications were graded using the Society of Interventional Radiology (SIR) classification (Spies et al., 2005).

### Statistical analysis

One sample t-tests of paired differences were used to test if the change in inflammatory markers, hormonal assays, Quality of Life scores, uterine volume and diameter of largest fibroid between baseline and 6 months post embolisation was significantly different from zero, for each treatment separately. Analysis of Covariance (ANCOVA) was applied to inflammatory markers, hormonal assays, Quality of Life scores, uterine volume and diameter of largest fibroid to test for a difference between treatments at 6 months post embolisation, after adjusting for baseline values. Pain score at 24 h, days till return to work and days till menses return were compared between groups using the Mann Whitney test.

## Results

Ten patients were randomized to each group and all received their allocated treatment. All procedures were technically successful with both uterine arteries embolised to complete stasis. All patients were followed up to 6 months. The groups were well matched at baseline (Table 2).

**Table 2:** Baseline clinical parameters for both groups

Parameter	Embospheres	Gelfoam
Median age in years (range)	42 (32–51)	45 (36–52)
Largest fibroid diameter (cm)	9.2 ± 3.8	9.5 ± 4.8
Uterine volume (cc)	1018 ± 666	1026 ± 756
UFS-Qol (symptom severity score)	64 ± 18	54 ± 15
UFS-Qol (HRQL score)	41 ± 28	53 ± 19

There were no significant differences between groups

### Embolic agent dose and radiation dosimetry

The mean weight of Gelfoam used was 0.225 g (range 0.06–0.53). In the Embosphere group 4 ampoules of 500–700 μm were given in all patients and a mean of 4 additional ampoules of 700–900 μm. One patient required 20 ampoules of Embospheres to achieve complete stasis.

Mean fluoroscopy time in G group was 20 min (range 11.1–31.3) with a mean radiation dose of 4204 cGym^2^ (range 846–14,004 cGym^2^). In the E group mean fluoroscopy time was 22.6 min (range 14.2–33.4) with a mean radiation dose of 5614cGym^2^ (range 1837–12,408 cGym^2^). There was no significant difference between groups. There were no catheter blockages reported in either group.

### Inflammatory markers and hormonal assays

WCC increased in both groups but remained within the normal range in the G group and only just outside normal range in the E group at 24-h (from 5.9 ± 2.2 at baseline to 12.3 ± 3.4 and from 6.4 ± 3.7 at baseline to 10.6 ± 2.6 in E and G groups respectively).

CRP increased at 24-h but remained within the normal clinical range at all time points in both groups. At 6 months these parameters had reduced to baseline levels (Table 3).

**Table 3:** Effect of embolic agent on inflammatory markers and hormonal assays

	Embospheres				Gelfoam				Difference between groups (E-G)	
	Baseline^a^	6 months^a^	Change^b^	p	Baseline^a^	6 months^a^	Change^b^	p	Difference^c^	p
WCC^1^	5.9 (2.2)	6.5 (1.8)	0.6 (−0.2 to 1.4)	0.11	6.4 (3.7)	6.3 (1.0)	−0.1 (−2.9 to 2.5)	0.90	0.4 (−0.9 to 1.7)	0.56
CRP^2^	1.4 (0.8)	2.1 (2.5)	0.9 (−0.7 to 2.5)	0.24	2.8 (2.6)	1.4 (1.0)	−1.3 (−2.6 to 0.1)	0.06	1.5 (−0.4 to 3.5)	0.12
LH^3^	8.0 (10.1)	11.5 (9.7)	3.5 (−2.6 to 9.6)	0.23	7.5 (9.4)	12.5 (15.9)	5.0 (−0.3 to 10.3)	0.06	−1.5 (−9.2 to 6.2)	0.68
FSH^4^	11.7 (13.3)	13.9 (12.6)	2.2 (−4.2 to 8.5)	0.46	11.5 (12.6)	21.4 (27.5)	9.9 (−3.2 to 23.0)	0.12	−7.7 (−21.4 to 5.8)	0.25
^5^E2^d^	135.5 (107.8308.0)	226.0 (73.3370.8)	^d^	^d^	213.0 (80.0,309.3)	129.5 (70.0,315.0)	^d^	^d^	^d^	^d^
^6^AMH^d^	4.0 (4.0,4.6)	4.0 (4.0,4.0)	^d^	^d^	4.0 (4.0,4.0)	4.0 (4.0,4.0)	^d^	^d^	^d^	^d^

^a^Mean (SD)

^b^Mean of change over time calculated as 6 month – baseline, 95% CI of change

^c^Mean difference between embolic agents at 6 months adjusted for baseline values, 95% CI of difference. Positive values indicate higher results in the E group

^d^Median (inter-quartile range). No analyses carried out for AMH and E2 due to large numbers of results recorded as `below limit of detection’

^1^Normal range for WCC is 4–11 × 10^9^/L. ^2^Normal CRP is less than 10 mg/L. ^3, 4^Normal range in pre-menopausal women for LH is 5 to 25 IU/L and FSH is 4.7–21.5 IU/L. ^5^Normal range for Estradiol (E2) in follicular phase is 77–920 pmol/L, mid cycle is 140–2380 pmol/L and luteal phase is 77–1145 pmol/L. ^6^Normal range in pre-menopausal women for AMH < 50 pmol/L

Individual patient hormonal profiles are shown in Table 4. FSH increased above the normal range in one patient in each group. In the E group from < 0.5 mIU/ml to 24.8 mIU/ml and in the G group from 17.1 mIU/ml to 57.9 mIU/ml. LH increased above the normal range in one patient in the G group from 11.3 IU/L to 26 IU/L. Serum estradiol (E2) levels dropped below normal range in 2 patients in each group (Table 4).

**Table 4:** Hormone levels at baseline and 6 months

No	Group	Age	LH		FSH		E2	
			Baseline	6 months	Baseline	6 months	Baseline	6 months
1	E	34	2.90	2.60	4.10	5.50	**287.00** ^c^	**83** ^c^
2	E	43	33.40	28.30	31.90	36.70	< 70	< 70
3	E	45	2.60	4.20	3.70	4.10	614.00	411
4	E	48	2.70	3.30	5.70	6.90	**315.00** ^c^	**< 70** ^c^
5	E	40	16.80	12.40	39.50	29.80	< 70	< 70
6	E	51	< 0.5	23.00	**< 0.5** ^b^	**24.80** ^b^	104.00	228
7	E	41	7.10	21.40	15.00	18.60	135.00	408
8	E	46	5.50	6.80	8.10	2.70	136.00	724
9	E	42	0.90	1.20	3.80	3.40	119.00	224
10	E	32	7.80	11.90	5.10	6.60	316.00	259
11	G	45	3.80	4.50	3.60	6.10	307.00	336
12	G	45	5.30	4.00	4.70	1.70	405.00	837
13	G	36	5.80	4.10	10.20	3.70	183.00	119
14	G	43	3.80	7.20	5.70	7.00	110.00	648
15	G	52	**11.30** ^a^	**26.00** ^a^	**17.10** ^b^	**57.90** ^b^	**243.00** ^c^	**< 70** ^c^
16	G	52	32.20	53.10	31.30	78.00	**420.00** ^c^	**< 70** ^c^
17	G	41	< 0.5	0.90	< 0.5	1.90	< 70	< 70
18	G	46	0.90	8.40	4.00	9.60	310.00	252
19	G	47	1.80	5.40	2.30	6.90	< 70	140
20	G	52	9.50	11.30	35.90	41.40	< 70	< 70

^a^Rise in LH level to menopausal range at 6 months in 1 patient

^b^Rise in FSH level to menopausal range at 6 months in 1 patient

^c^Estradiol levels dropped below normal range at 6 months in 4 patients

Normal range in pre-menopausal women for LH is 5 to 25 IU/L and FSH is 4.7–21.5 IU/L

Normal range for Estradiol (E2) in follicular phase is 77–920 pmol/L, mid cycle is 140–2380 pmol/L and luteal phase is 77–1145 pmol/L

In the G group one patient age 52y had a large rise in FSH, LH and a drop in E2 however menses returned at 6 months. In the E group menses did not return in two patients (age 40, 51). One of these patients age 40 had persistently high FSH at baseline (39.5 mIU/ml) and 6 months (29.8 mIU/ml). Further follow-up would be required to determine any long-term effect on ovarian function.

AMH levels were reported as ‘less than detectable levels’ in 14 patients at baseline, 12 at 24 h post-embolisation, 15 at 1 month and 18 patients at 6 months. Results were not available for 3 patients at 24 h post-embolisation. Hence statistical analyses could not be performed for AMH levels.

### Quality of life and symptom score (UFS-QoL)

The UFS-Qol symptom severity score reduced in both groups at 6 months, from a mean of 64 ± 18 to 23 ± 16 and 54 ± 15 to 32 ± 26 in the E and G groups respectively. UFS-Qol HRQL score improved in both groups at 6 months, from a mean of 41 ± 28 to 79 ± 20 and 53 ± 19 to 78 ± 21 in the E and G groups respectively (Table 5).

**Table 5:** Effect of embolic agent on UFS-QoL symptom score and HRQL score

	Embospheres			Gelfoam			Difference between groups (E-G)***
	Baseline*	6 months*	Change**	Baseline*	6 months*	Change**	
Symptom score	64 (18)	23 (16)	−41 (−56 to − 25) *p* < 0.001	54 (15)	32 (26)	−22 (− 36 to − 8) *p* = 0.01	−14 (− 34 to 6) *p* = 0.15
HRQL	41 (28)	79 (20)	38 (13 to 63) *p* = 0.01	53 (19)	78 (21)	25 (9 to 41) *p* = 0.01	2 (−18 to 22) *p* = 0.84

*Mean (SD)

**Mean of change over time calculated as 6 month – baseline, 95% CI of change, *p*-value

***Mean difference between embolic agents at 6 months adjusted for baseline values, 95% CI of difference. Positive values indicate higher results in the E group

There was no significant difference in symptom severity or HRQL between the two groups at 6 months (*p* = 0.15 and *p* = 0.84 respectively) after adjusting for baseline values (Table 5).

### Complications and re-interventions

In the G group there was one minor (grade 1) and one major complication (grade 3). The minor complication was a groin haematoma and the major complication a pelvic infection (which required hospitalization for antibiotic therapy). In the E group there were 4 minor complications (two urinary tract infections and 2 fibroid expulsions). None of the patients required any invasive re-interventions for either complications or persistent or recurrent symptoms at final 6 month follow up.

### Pain scores, return to work and return of menses

Median pain scores at 24 h were marginally higher in the E group 3 (IQR = 2–3.8) versus 2 (IQR = 1.3–2) in the G group (*p* = 0.07). Median time to return to work was 15 (IQR = 11–21) days in the E group and 21 (IR = 15–23) days in the G group (*p* = 0.19). There was no statistically significant difference between groups for either measure.

By 6 months menses had returned in all patients in the G group but only 8 in the E group. The median time for menses to return was 28 (22.3–42.5) days in the E group and 22 (19–25) days in the G group. These differences were not significant (*p* = 0.31).

### Imaging outcomes

There was a significant reduction in uterine volume in the E group but not in the G group. The mean uterine volume reduced from 1018 ± 666 cc at baseline to 622 ± 436 cc at 6 months (*p* = 0.001) in the E group. In the G group the volume reduced from 1026 ± 756 cc at baseline to 908 ± 720 cc at 6 months (*p* = 0.15). There was a significant difference between groups at 6 months (*p* = 0.01) (Table 6).

**Table 6:** Effect of embolic agent on uterine volume, diameter of largest fibroid, infarction rate and uterine artery patency

	Embospheres			Gelfoam			Difference between groups (E-G)***
	Baseline*	6 months*	Change**	Baseline*	6 months*	Change**	
Uterine volume (cc)	1018 (666)	622 (436)	− 257 (− 398 to − 115) *p* = 0.001	1026 (756)	908 (720)	− 117 (− 286 to 52) *p* = 0.15	−281 (− 496 to − 66) *p* = 0.01
Largest fibroid diameter (cm)	9.2 (3.8)	7.3 (3.4)	− 2.0 (− 3.0 to − 1.0) *p* = 0.002	9.5 (4.8)	8.8 (5.9)	−0.7 (− 1.9 to 0.5) *p* = 0.20	−1.3 (− 2.7 to 0.2) *p* = 0.09
Uterine artery		10 (100%)			10 (10%)		
Complete infarction (100%)		5 (50%)			2 (20%)		
Almost complete infarction (99–90%) 99–90%)		3 (30%)			1 (10%)		
Incomplete infarction (< 90%)		2 (20%)			7 (70%)		

*Mean (SD)

**Mean of change over time calculated as 6 month – baseline, 95% CI of change, *p*-value

***Mean difference between embolic agents at 6 months adjusted for baseline values, 95% CI of difference. Positive values indicate higher results in the E group

Similarly there was a significant reduction in dominant fibroid diameter in the E group but not in the G group. The mean dominant fibroid diameter reduced from 9.2 ± 3.8 cm to 7.3 ± 3.4 cm (*p* = 0.002) in the E group and from 9.5 ± 4.8 cm to 8.8 ± 5.9 cm (*p* = 0.20) in the G group at 6 months. There was no significant difference between groups (*p* = 0.09) at 6 months (Table 6). Infarction rates are shown in Table 6, complete infarction of the entire fibroid tissue at 6 months was achieved in 5(50%) and 2(20%) in the E and G groups respectively (ns). All uterine arteries were found to be patent at 24-h and 6 months in both groups.

## Discussion

This randomized trial comparing Gelfoam with Embospheres was intentionally designed as a pilot study as there was insufficient data in the literature on which to base a power calculation for a definitive study. The only significant difference between the two groups was in the reduction of uterine volume at 6 months which amounted to 281cm^2^ in favour of Embospheres (*p* < 0.01). Furthermore the reduction in uterine volume between baseline and 6 months was significant in the E group (1018 cm^2^ to 622 cm^2)^ (*p* = 0.001) but non-significant in the G group (1026 cm^2^ to 908 cm^2^) (*p* = 0.15). This was matched by a significant reduction in dominant fibroid diameter in the E group (*p* = 0.002) but not in the G group (*p* = 0.2). There was no significant between group differences with regard to dominant fibroid diameter (*p* = 0.09). For the remaining outcome measures we found no significant between-group differences. The clinical significance of the difference in uterine volume reduction is unclear. Although quality of life and symptom score improved significantly in both groups from baseline there was no significant difference between groups. Similarly the re-intervention rate (which was zero at 6 months) did not vary between groups although it could be argued that the follow up period was too short for this outcome measure.

Ovarian function following UAE is difficult to assess. Even when menses do not return it is almost impossible in women aged >40y to determine whether this is due to the natural ageing process or the UAE procedure. We had hoped AMH levels would provide some answers as this hormone is a surrogate marker for the total follicle count indicating ovarian reserve and is independent of the menstrual cycle day. However although useful in younger women <40y the current assay available was unable to detect any measurable levels in 14 of the 20 patients at baseline and in 18 of the 20 patients at 6 months. The mean age was 42y and 45y in the E group and G group respectively. Menses had not resumed in 2 patients in the E group by 6 months; these two patients were aged 40y and 51y. Menses had resumed in all patients in the G group at this time point. Interpretation of these small numbers is difficult and further follow-up is required to determine any long-term effect on ovarian function from either agent.

There have been 5 randomised trials published to date and all have compared Embospheres with spherical PVA (3 trials), non-spherical PVA (1 trial) or Beadblock (1 trial) (Spies et al., 2004; Siskin et al., 2008; Worthington-Kirsch et al., 2011; Yu et al., 2011; McPherson et al., 2014). There have been no comparative studies with Gelfoam. A recent systematic review and meta-analysis of these trials found no superiority of any embolic agent over another (Moss et al., 2011). Although the outcome measures across these trials varied there was reasonable information on UFS-QOL scores and uterine and fibroid volumes. When the two trials comparing Embospheres with spherical PVA were specifically combined the authors found a trend towards greater uterine and dominant fibroid volume reduction with Embospheres. Embospheres also demonstrated greater fibroid devascularisation than spherical PVA. It should be noted that spherical PVA is no longer available as an embolic agent. The systematic review stated that the comparison between agents was hampered by a lack of RCT data and encouraged further research (Moss et al., 2011). There are 4 other trials underway which were identified in the systematic review including our pilot trial.

This study has several limitations. Firstly there were only 20 patients in total but from the outset the design was a pilot trial. Secondly there was no subjective assessment of menstrual loss, for example using a pictorial menstrual diary. The decision not to use one was based on our previous experience of poor compliance with these diaries. Thirdly the patient group was fairly old (mean age > 40y) and AMH proved an ineffective assay for most of the patients. Refinements in this assay may hold some hope for the future but at present it seems only to be useful in women aged <40y which is the age group of most concern as they tend to wish to maintain fertility. A larger cohort (*n* = 250) is currently under investigation in the U.K. FEMME trial which is due to report in 2019 (Macnaught et al., 2016). Finally follow up was short and it is well recognized that there is a significant re-intervention rate beyond this time window out to 5 years and perhaps beyond (Moss et al., 2011).

## Conclusion

In conclusion we were only able to demonstrate a significance difference between these two embolic agents with regard to uterine volume reduction. The clinical significance of this is unclear and was not reflected in any difference in quality of life or any other outcome measures. All the other parameters including haematological, inflammatory and ovarian function showed no differences together with fibroid infarction and uterine artery patency rates. There were no differences in radiation penalty, quality of life or other clinical parameters. Further research is clearly needed in this area and to detect a difference in mean UFS-QOL symptom score of 0.5 standard deviation with 90% power would require a trial of at least 172 patients, 86 in each arm.

## Abbreviations

AMH: Antimullerian hormone
E: Embospheres
FSH: Follicular stimulating hormone
G: Gelfoam
LH: Luteinizing hormone
PVA: Polyvinyl alcohol
UAE: Uterine artery embolization
